# Microstructure Heredity and Phase Transformation of CoFeB Pre-Alloyed Powder During Hot Pressing Sintering

**DOI:** 10.3390/ma19163418

**Published:** 2026-08-12

**Authors:** Zehua Ren, Qian Jia, Junfeng Luo, Xinran Li, Zhaochong Ding, Yutong Ran, Jinjiang He

**Affiliations:** 1 Grikin Advanced Materials Co., Ltd., Beijing 102200, China; ren841692676@163.com (Z.R.); jq@grikin.com (Q.J.); ljf@grikin.com (J.L.); lixinran@grikin.com (X.L.); dzc@grikin.com (Z.D.); 2 National Engineering Research Center of Key Materials of Integrated Circuit, Beijing 100088, China; 3 General Research Institute for Nonferrous Metals, Beijing 100088, China

**Keywords:** Co_40_Fe_40_B_20_ soft magnetic alloy, gas atomization, hot pressing sintering, powder particle size, phase transformation, microstructure evolution

## Abstract

The Co_40_Fe_40_B_20_ alloy is a key magnetic material that combines high saturation magnetization with excellent soft magnetic properties, offering broad application prospects in fields such as spintronic devices, magnetic tunnel junctions, and tunnel magnetoresistive sensors. Hot pressing can be used to produce fine-grained, highly dense CoFeB alloys. However, there is currently a lack of systematic research on the intrinsic mechanisms by which the particle size of gas-atomized CoFeB powders and their non-equilibrium solidification microstructure regulate phase transformations, microstructural evolution, and densification behavior during hot pressing and sintering—particularly regarding the microstructural inheritance effects of powders with different particle sizes. To address this issue, this study used vacuum induction melting and gas atomization technology to prepare Co_40_Fe_40_B_20_ pre-alloyed powders in three particle size ranges: <38 μm, 38–74 μm, and 74–154 μm. Under identical process parameters, corresponding bulk alloys were produced via vacuum hot-press sintering, and the effects of initial powder particle size on phase transformations and microstructural evolution in the sintered bodies were systematically investigated. Microstructural characterization revealed the complete phase evolution of the alloy from the non-equilibrium solidified powder state to the sintered equilibrium state. During hot-press sintering, the metastable (Fe,Co)_3_B phase in the powder completely decomposed, transforming into a stable body-centered cubic bcc-(Fe,Co) phase and a bcc-(Fe,Co)_2_B second phase. The dispersed (Fe,Co)_2_B phase precipitated after sintering strongly inhibits grain boundary migration via the Zener pinning effect, effectively hindering grain growth and resulting in a uniform, fine-grained, equiaxed microstructure. In coarse powders, due to the presence of a portion of the (Fe,Co)_2_B phase, this phase aggregates and grows during sintering, weakening the pinning effect and leading to abnormal grain growth. The Hall–Petch fine-grain strengthening effect resulting from grain refinement couples with and offsets the weakening of second-phase strengthening caused by second-phase coarsening, ultimately leading to sintered bodies prepared from powders of different particle sizes exhibiting similar macroscopic density and hardness properties.

## 1. Introduction

As an important class of soft magnetic materials, CoFeB exhibits high saturation magnetization, excellent soft magnetic properties, and good magnetic permeability, demonstrating broad application prospects in fields such as spin devices, magnetic tunnel junctions, and tunnel magnetoresistance sensors [1,2,3]. Particularly given the current trends toward miniaturization and higher frequencies in electronic devices, optimizing the performance of CoFeB materials and improving their fabrication processes have become key areas of focus in materials science research [4,5,6].

Vacuum induction–melting gas atomization (VIGA) is a key method for producing high-purity multi-component alloy powders. It involves using a high-pressure, high-velocity argon gas stream to impact a molten metal stream, breaking it into fine droplets that rapidly cool and solidify to form spherical powders [7,8,9]. During the solidification process, the nucleation driving forces of different phases in the CoFeB melt vary significantly, leading to distinct phase distribution characteristics. These variations give rise to unique driving forces during hot-press sintering. Powders with different phase compositions and microstructures exhibit varying sintering driving forces, which play distinct roles in the sintering densification process and have a significant impact on CoFeB materials that are difficult to densify [8,10,11,12].

Currently, there remains a lack of research on the influence of CoFeB powders on the sintering densification process and the evolution of microstructure during sintering. In particular, the markedly different microstructural evolution behaviors exhibited by coarse-grained powders (150 μm) and fine-grained powders (38 μm or smaller) during hot-press sintering, as well as the resulting differences in densification mechanisms, require in-depth theoretical analysis and experimental verification. This study aims to reveal the influence of the phases in CoFeB gas-atomized powders on the driving forces of sintering through systematic experimental research and theoretical analysis, thereby providing a scientific basis for the preparation of highly dense CoFeB materials.

## 2. Experiment

In this study, CoFeB soft magnetic alloys were prepared using high-purity elemental ingots as raw materials, with the Co ingot having a purity of ≥99.99%, the Fe ingot having a purity of ≥99.99%, and the B ingot having a purity of ≥99.99%. The target alloy composition is Co_40_Fe_40_B_20_ (atomic percentage, at.%), which represents a classic CoFeB soft magnetic system characterized by excellent amorphous formation capability, high saturation magnetization, and low coercivity.

Co_40_Fe_40_B_20_ alloy powder was prepared using vacuum induction melting and gas atomization equipment. The core process is as follows:

Co, Fe, and B ingots, weighed according to the specified ratio, were placed in an alumina crucible. After sealing the melting chamber, a vacuum was first evacuated, followed by back-filling with high-purity argon to a slight positive pressure to prevent oxidation of the alloy during melting. The induction power supply is activated to begin melting. Once the melt is fully liquefied and the composition is homogeneous, high-purity argon gas is used as the atomization medium. A high-pressure gas stream breaks up the molten alloy into fine droplets, which rapidly cool and solidify into spherical alloy powders within the atomization tower. Collect the atomized powder and grade it using standard test sieves in a glove box protected by high-purity argon gas. This yields powder samples in three different particle size ranges: less than 38 μm (below 400 mesh, designated as fine powder), 38–74 μm (200–400 mesh, designated as medium powder), and 74–154 μm (100–200 mesh, designated as coarse powder). Three powder samples were each uniformly blended with a conductive graphite hot-mounting resin, processed into blocks by hot mounting, and then ground and polished to expose cross sections of the powders for analysis. The sieved powders and blocks are stored in vacuum-sealed containers to prevent oxidation and contamination.

Co_40_Fe_40_B_20_ bulk alloys were prepared using a vacuum hot-press sintering furnace. A systematic study was conducted to investigate the effects of powder particle size on the densification behavior of the sintered bodies. The experiments followed the single-variable principle, with all sintering process parameters kept identical except for the powder particle size. The specific preparation process is as follows:

A high-purity graphite mold with an inner diameter of 75 mm and a matching graphite punch were used. After the graphite mold was thoroughly dried, 200 g of the powder to be sintered was weighed and loaded into the mold within a glove box under a high-purity argon atmosphere. The powder-filled mold was then placed into the vacuum hot-press sintering furnace. After sealing the furnace, a vacuum was evacuated. When the temperature reached 900 °C, the system was held at that temperature and pressure. The heating rate was controlled within the range of 5–20 °C/min, and the isothermal holding time at 900 °C was between 60 and 180 min. Upon completion, the sample was cooled in the furnace to room temperature to reduce residual thermal stress in the sintered body. After removing the green body, the surface-attached graphite and oxide scale were removed by wire cutting, resulting in a clear block-shaped alloy sample with dimensions of 10 mm × 10 mm × 15 mm. For each particle size range, three parallel samples are prepared under identical process conditions. All performance tests are conducted separately on the three parallel samples, and the results are averaged to calculate the relative standard deviation, ensuring the reliability and reproducibility of the experimental data.

The 10 mm × 10 mm × 15 mm block-shaped sintered sample was fixed with epoxy resin to ensure stable operation. A stepwise grinding process was conducted using silicon carbide sandpapers with grit sizes of 240#, 400#, 800#, 1200#, 2000#, and 3000# in sequence, with each grinding step sustained until scratches from the previous step were fully eliminated. Afterwards, a two-stage polishing procedure was performed: rough polishing was carried out on a nylon polishing cloth with 2.5 μm diamond suspension to remove residual grinding scratches, followed by fine polishing on a velvet polishing cloth using 0.5 μm diamond suspension and final polishing with 0.05 μm colloidal silica suspension to achieve a mirror-like, scratch-free surface. The polished samples were then ultrasonically cleaned in anhydrous ethanol for 5 min and blown dry with high-purity nitrogen prior to microstructure characterization.

The bulk density of the sintered specimens was measured by the Archimedes immersion method using a high-precision electronic analytical balance. Each specimen was tested for no less than three parallel times to ensure data reliability, and the relative density was calculated by comparing the measured density with the theoretical density of the Co_40_Fe_40_B_20_ alloy.

For the hardness characterization, Vickers hardness tests were performed on a digital Vickers hardness tester under a test load of HV1 with a dwell time of 15 s. Ten valid indentation points were randomly selected on the polished cross-section of each sample to calculate the average hardness value, so as to eliminate the interference of local microstructural inhomogeneity on the test results.

The microhardness of the specimens was measured using a Vickers hardness tester (model: HVS-1000, Shanghai Jvjing, Shanghai, China) with a load of 200 gf and a dwell time of 15 s. The particle size distribution of the powders was determined by a laser diffraction particle size analyzer (model: Mastersizer 3000, Malvern Panalytical, Malvern, UK) employing the wet dispersion method. A scanning electron microscope (SEM, EBSD; model: JSM-IT800, JEOL, Akishima, Japan) was used to examine the morphology of the alloy powders and sintered specimens, as well as the grain size and phase distribution of both the powders and the specimens. An X-ray diffractometer (XRD; model: D8 DISCOVER, Bruker, Billerica, MA, USA) was used to perform phase analysis on the powders and sintered specimens. The test conditions were as follows: Co target Kα radiation (λ = 0.1789 nm), tube voltage 35 kV, tube current 30 mA, scan range 2θ = 5–90°, step size 0.02°, and continuous scan rate 2°/min. A transmission electron microscope (TEM, JEM-2100F, JEOL) was used to study phase transformations in the sintered bodies. After mechanical thinning of the samples to 50 μm, TEM specimens were prepared using an ion mill (Gatan 691, Pleasanton, CA, USA). The phase structure was analyzed using selected-area electron diffraction (SAED), and grain transformation features were observed via high-resolution TEM (HRTEM).

## 3. Results and Discussion

**Figure 1:**
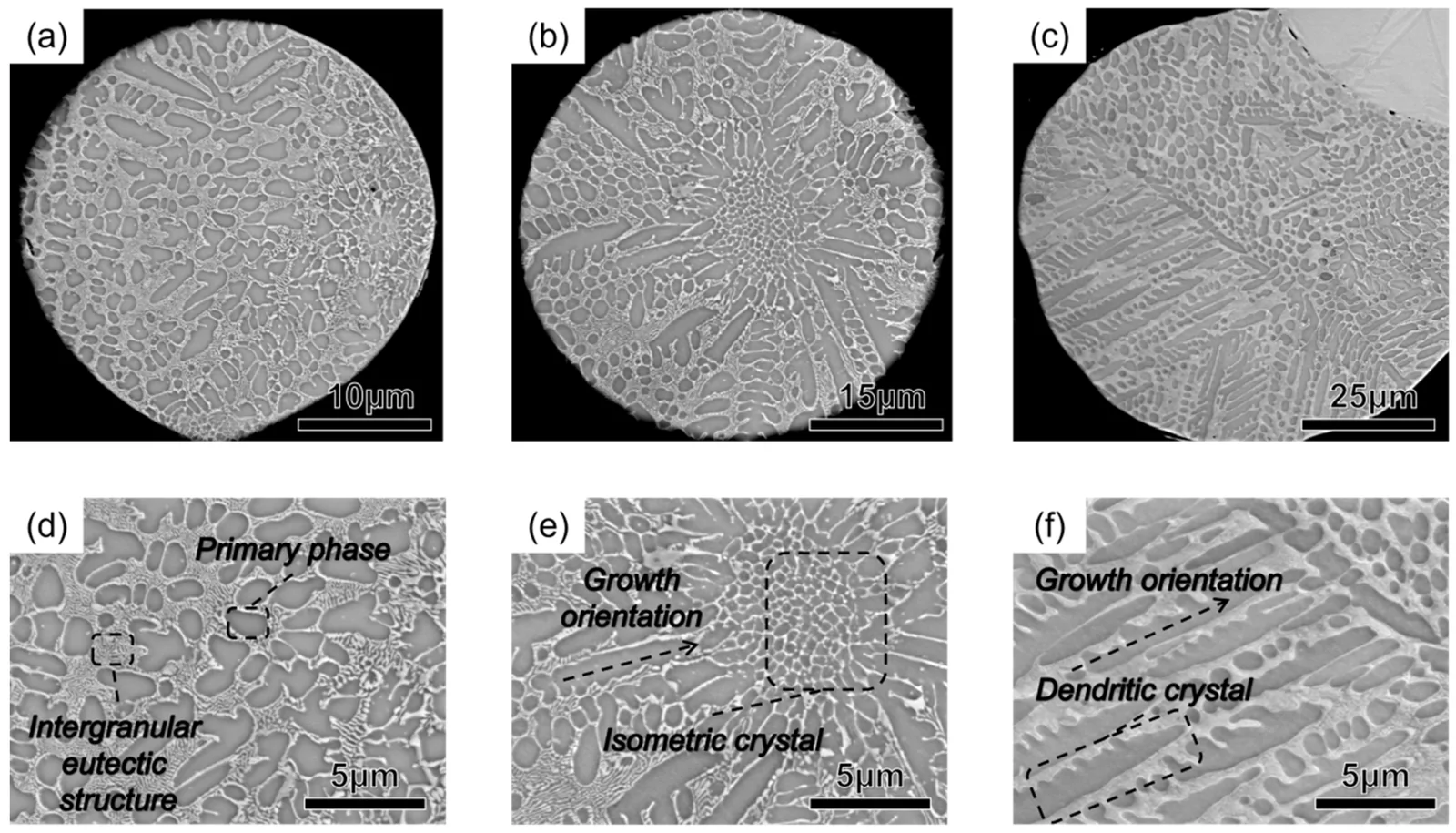
Cross-sectional views of powders with different particle sizes. (**a**) Fine powder, (**b**) medium powder, (**c**) coarse powder; (**d**–**f**) are magnified views of the corresponding regions.

The microstructure within the powder has a certain influence on the sintered body; characteristics such as grain size and phase distribution are inherited [13,14,15]. Cross-sections of powder samples with different particle sizes were characterized, as shown in Figure 1. Figure 1a shows a cross-section of the fine powder. Due to the small particle size (less than 38 μm), the cooling rate is rapid. The cross-section exhibits a typical cast-like, non-equilibrium solidification microstructure characteristic of rapid cooling. During the gas atomization process, the melt cools after forming into spheres, resulting in ellipsoidal or equiaxed primary phases (boride phases). After the primary phase forms, the temperature continues to decrease, leading to the formation of a fine, network-like structure around it, known as the eutectic structure ((Fe,Co) solid solution and boride phase). Ultimately, this results in a structure with a dispersed primary phase and an intergranular eutectic structure.

As the powder particle size increases, the cross-sectional microstructure of the powder develops distinct radially extending dendritic crystals and a central zone of fine equiaxed grains. The spaces between the dendritic crystals consist of a dense, mesh-like eutectic structure, resulting in a typical cast microstructure characterized by directional heat dissipation, as shown in Figure 1b. When the powder particle size was further increased to 74–154 μm, as shown in Figure 1c, the powder volume increased, and the heat dissipation rate exhibited differences. On one side, radial dendritic crystals formed; due to the lower cooling rate, primary phase growth was sufficient, and no eutectic structure was formed; instead, a two-phase structure composed of the primary phase and the solid solution phase was formed. On the other side, due to the increased cooling rate, a transition zone from columnar grains to equiaxed grains appears. In this transition zone, the grain orientation tends toward an ellipsoidal shape, with a eutectic structure distributed between the grains and localized coarsening of the primary phase.

Due to differences in phase-driving forces, the boride phase forms first during powder formation. As the boron concentration around the primary phase decreases—that is, as the boride phase forms preferentially in the melt—the remaining melt forms the (Fe,Co) phase. As shown in Figure 1a, powders with particle sizes <38 μm consist of equiaxed grains of varying diameters, while powders in the 73–150 μm range exhibit a cross-sectional structure characterized by coarse dendritic crystals growing from the periphery toward the center and a secondary eutectic phase between the dendrites. Powders in the 38–74 μm range exhibit a transitional microstructure, with grain sizes and morphologies intermediate between the two—that is, transitional characteristics between fine equiaxed grains and coarse dendritic eutectic structures.

**Figure 2:**
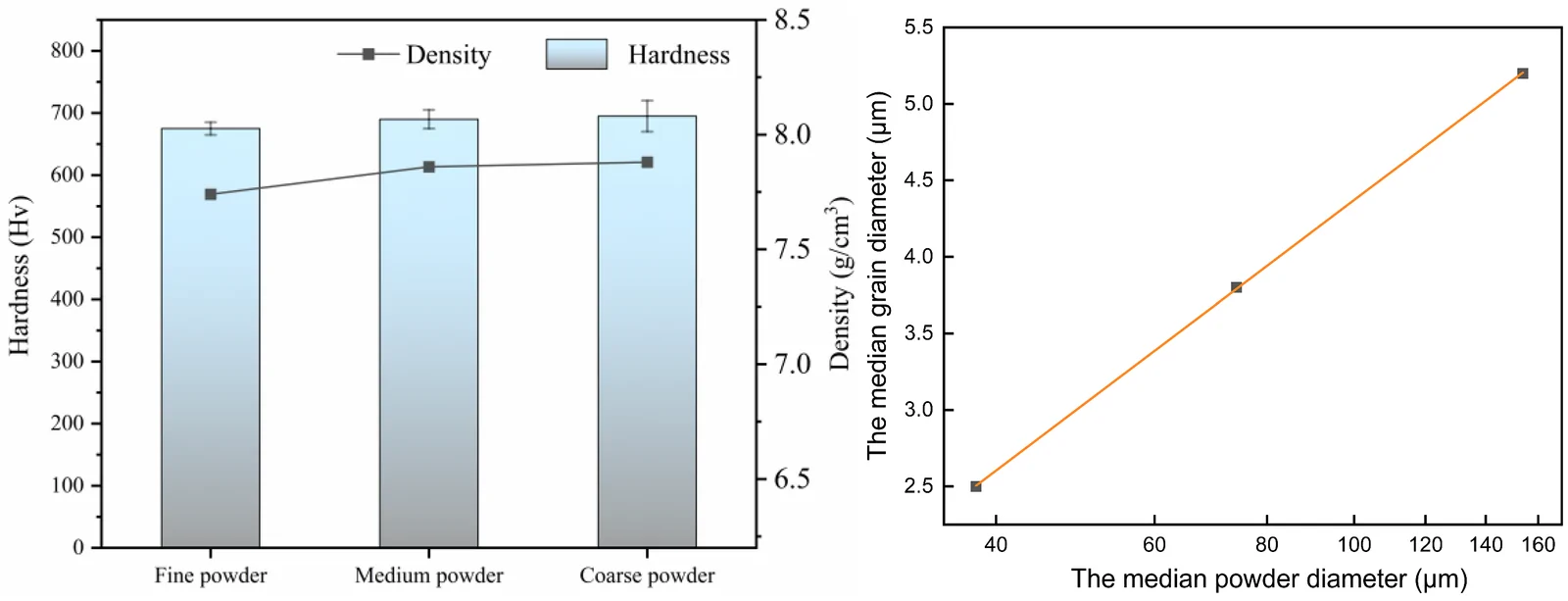
Density, hardness and grain diameter of sintered compacts with different initial powder particle sizes.

The sintered bodies prepared from CoFeB powders of different particle sizes exhibited similar densities and Vickers hardness values, with no significant differences. Specifically, the densities of the sintered bodies made from fine, medium, and coarse powders were 7.85 g/cm^3^, 7.86 g/cm^3^, and 7.88 g/cm^3^, respectively, with a maximum difference of only 0.03 g/cm^3^ and a relative deviation of less than 0.4%. The corresponding Vickers hardness values were 682 HV, 689 HV, and 683 HV, with a maximum difference of only 7 HV and a relative deviation of approximately 1.0% (Figure 2). It can be seen that under the same hot-press sintering process, the densification process for powders of different particle sizes has been completed, and the hardness and density of the sintered bodies are similar. Therefore, the influence of surface activity factors caused by powder particle size on the sintering densification process is relatively minor.

As the particle size of the powder decreases, the half-width at half-maximum of the diffraction peaks continues to increase, the peak shapes gradually broaden, and the peak intensities decrease significantly [3,16,17,18,19,20,21,22,23,24]. According to the Scherrer formula, the smaller the grain size, the more pronounced the broadening effect of the diffraction peaks; consequently, as the particle size of the powder decreases, the grain size also decreases. According to phase diagram studies [6,8,25,26,27], during equilibrium solidification, the (Fe,Co)_2_B phase in borides exhibits the greatest nucleation driving force and is preferentially precipitated as the primary phase. However, during the VIGA process, smaller alloy droplets cool rapidly and exhibit a high degree of solidification undercooling. The rapid rate of temperature decline hinders the migration of boron atoms, making it difficult to form the (Fe,Co)_2_B phase, which requires the aggregation of a larger number of boron atoms. This leads to the precipitation of the (Fe,Co)_3_B phase, which has a lower boron content, while the remaining boron forms a supersaturated solid solution in the bcc-(Fe,Co) matrix rather than a boride phase.

**Figure 3:**
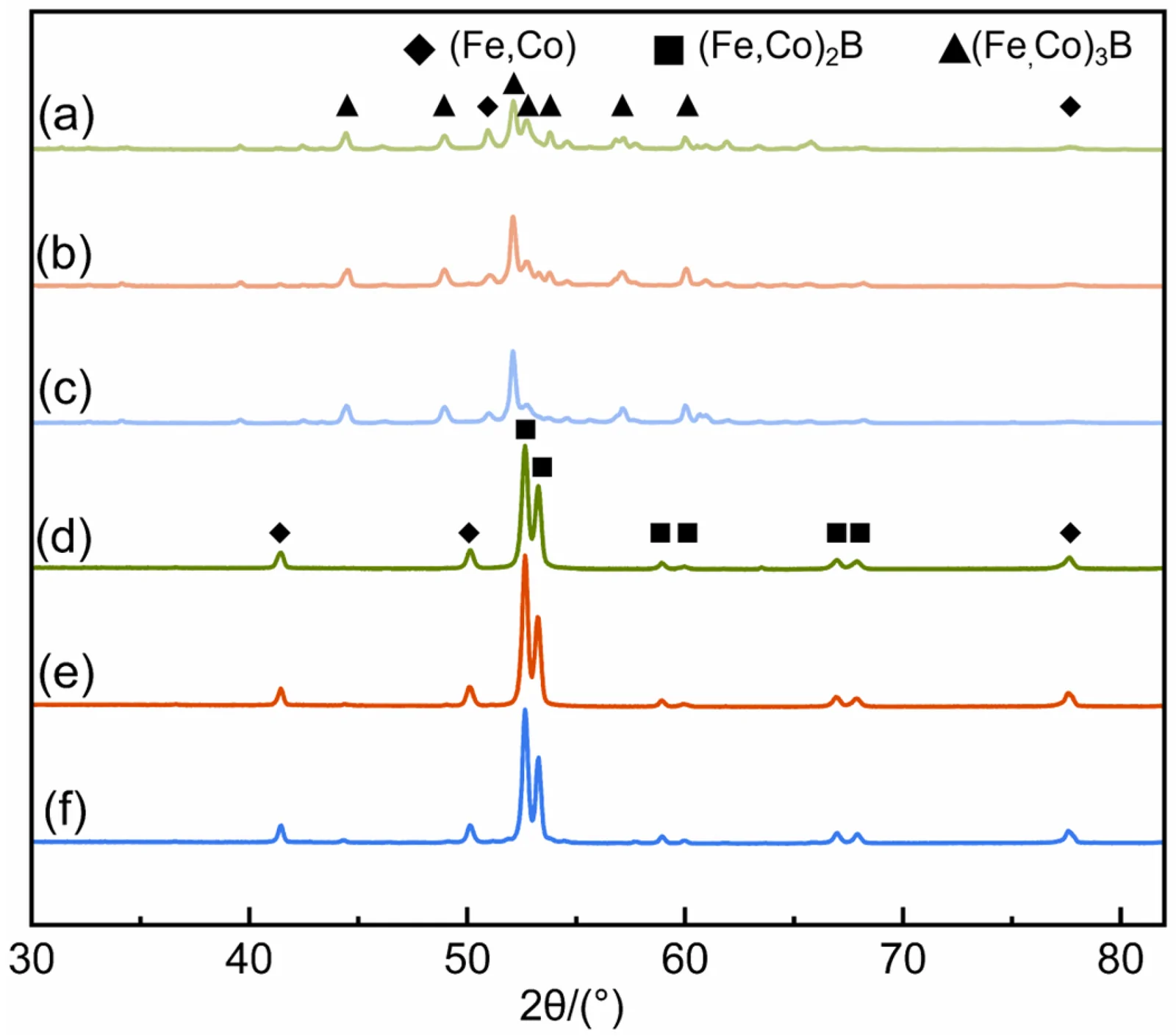
XRD patterns of powders with different particle sizes and the corresponding sintered compacts. (a) Fine powder; (b) medium powder; (c) coarse powder; (d) fine powder sintered compact; (e) medium powder sintered compact; (f) coarse powder sintered compact.

Among these, the fine powder shown in Figure 3(a) exhibits the most pronounced broadening effect, with the diffraction peaks of the (Fe,Co) phase displaying distinct broadening characteristics and clear features of a supersaturated solid solution.

As the particle size increases, the diffraction peaks of the (Fe,Co) phase become relatively sharper, the proportion of the supersaturated solid solution decreases, and the diffraction peak characteristics of the boride phase become more pronounced [14,28,29,30,31].

The diffraction peaks corresponding to the sintered body are all sharp, symmetrical, and distinct, with significantly narrowed half-widths, indicating that grain growth has occurred, internal stresses have been released, the decomposition of the supersaturated solid solution is complete, and the metastable phase (Fe,Co)_3_B has undergone complete decomposition:

(1) $${\left(Fe,Co\right)}_{3}B\to {\left(Fe,Co\right)}_{2}B+(Fe,Co)$$

The decomposed (Fe,Co) phase serves as the matrix, within which boron further enriches and grows, forming a stable (Fe,Co)_2_B phase in the (Fe,Co) matrix, thereby achieving a complete transformation of the boride phase.

In the XRD patterns, the positions of the (Fe,Co) phase diffraction peaks are consistent across all sintered bodies. Co atoms are fully solid-solved in the matrix, forming an additional diffraction peak shifted toward larger angles at the original peak positions, and the crystal structure does not undergo any fundamental changes due to these differences. After vacuum hot-press sintering of powders with different particle sizes, high temperatures provided sufficient kinetic conditions for atomic diffusion, enabling the sintered bodies to complete the densification process while also achieving a complete transition from a non-equilibrium state to an equilibrium state.

**Figure 4:**
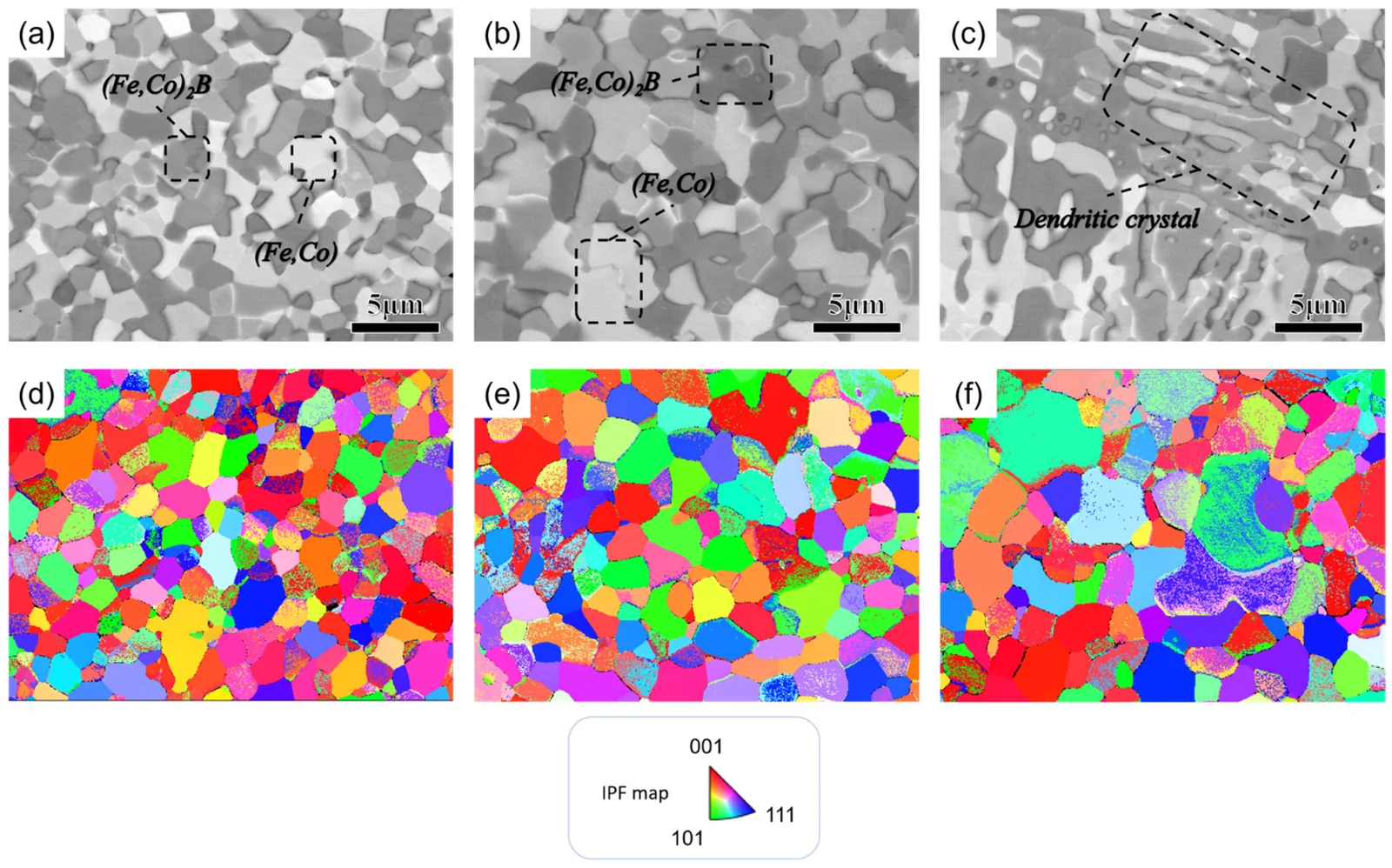
SEM and EBSD images of sintered bodies with different powder particle sizes. (**a**,**d**) fine-powder sintered body; (**b**,**e**) medium-powder sintered body; (**c**,**f**) coarse-powder sintered body.

The BSE images of sintered bodies made from CoFeB powders with different initial particle sizes clearly distinguish the distribution of the two phases. The light-colored phase represents the (Fe,Co) phase, while the dark-colored phase represents the (Fe,Co)_2_B phase, as shown in Figure 4 [7,22,32,33,34]. All three sets of sintered bodies are generally dense, with only a very small number of isolated micron-scale closed pores (black dot-like defects) and no interconnected pores, which is highly consistent with the density test results described earlier.

The microstructure of the sintered body made from fine powder exhibits a uniform, two-phase dispersion. The (Fe,Co) alloy has fine, uniformly distributed grains with an average grain size of approximately 1.2–1.8 μm and well-defined, intact grain boundaries. (Fe,Co)_2_B appears as nearly equiaxed, short rod-like particles uniformly distributed at the grain boundaries of the (Fe,Co) matrix and at the triangular grain boundaries. Some fine boride particles are dispersed within the matrix grains; their particle sizes range from 0.5 to 1.2 μm, and no continuous network structure is observed. The uniformly dispersed boride phase effectively suppresses matrix grain boundary migration and grain growth during sintering via the Zener pinning effect, resulting in excellent overall microstructural uniformity.

The average grain size of the medium-powder sintered body increases slightly to approximately 1.8–2.5 μm, with good grain size uniformity maintained; only a small number of grains exhibit a slight tendency toward growth, and the grain boundaries remain straight and distinct. The (Fe,Co)_2_B particle size increased slightly, concentrated in the 0.8–1.5 μm range, and remained predominantly dispersed. However, a slight tendency toward continuous distribution appeared at the grain boundaries of some adjacent matrix grains. No complete network structure was formed, and effective pinning of the matrix grain boundaries was still maintained. The overall microstructure retained good characteristics of uniform two-phase distribution, with no obvious phase agglomeration or segregation.

The sintered body of the coarse powder inherits the powder microstructure, retaining some characteristics of dendritic crystals. Locally, abnormally large grains exceeding 8 μm in size appear, with curved and irregular grain boundaries. In certain areas, distinct boride phase agglomerations form sheets, resulting in a significant decline in microstructural uniformity and a pronounced biphasic segregation pattern.

**Figure 5:**
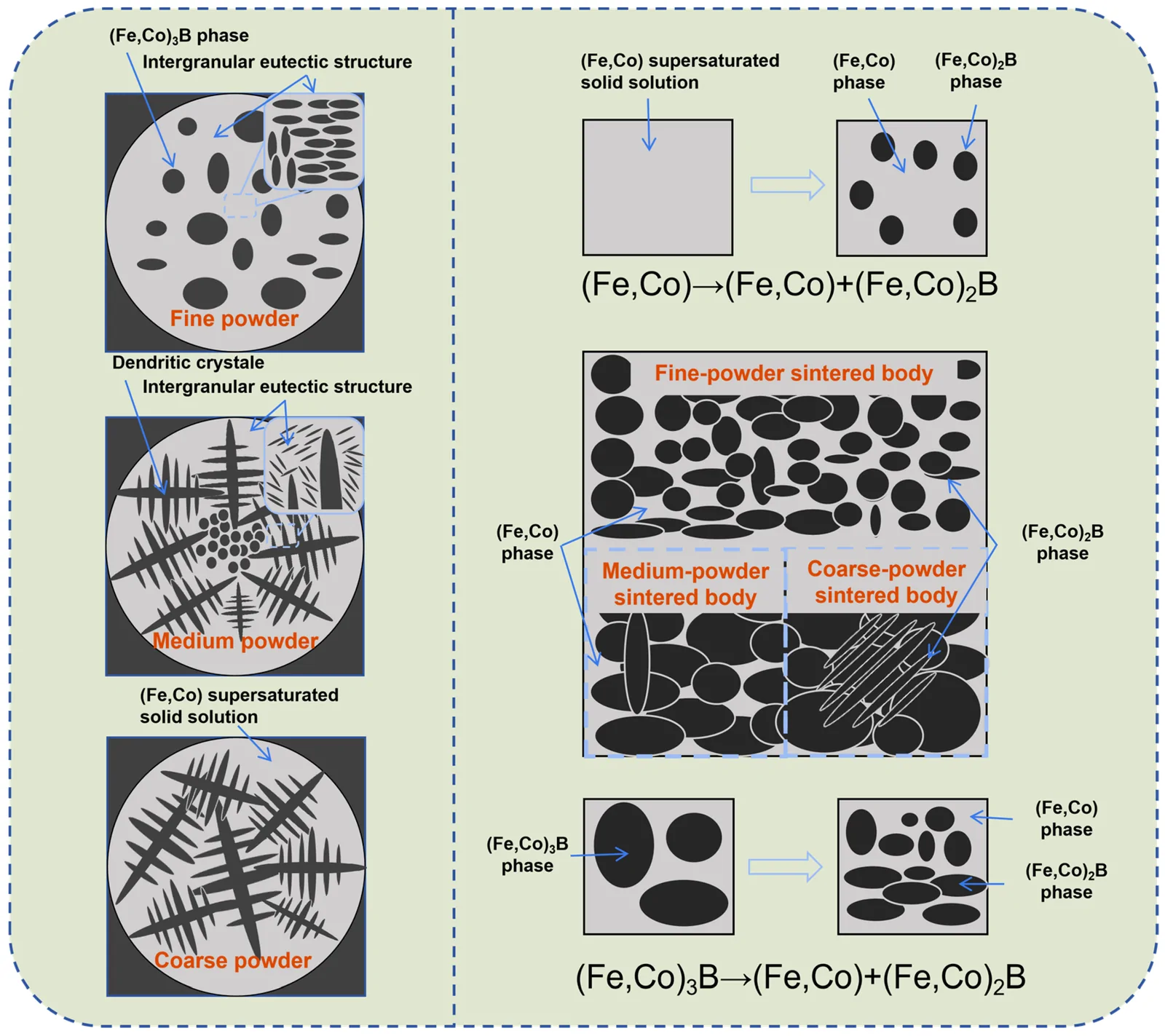
Powder microstructure and sintering mechanism.

The cooling rate of gas-atomized powders is inversely proportional to particle size. Fine powders cool rapidly, resulting in a high degree of solidification undercooling. This leads to a higher degree of supersaturation of boron (B) in the (Fe,Co) matrix. Consequently, the (Fe,Co)_2_B phase precipitated during sintering is more dispersed and abundant, exerting a stronger pinning effect on the matrix grain boundaries and effectively suppressing grain growth. Coarse powders cool slowly, resulting in low initial boron supersaturation (Figure 5). After sintering, the boride phase precipitates in smaller quantities and larger sizes, causing significant grain growth and localized abnormal growth in individual grains during the sintering holding phase, while retaining some of the powder’s dendritic characteristics. Although fine powders possess better surface activity, which is more conducive to sintering densification and grain growth, the presence of the second phase inhibits grain growth. Conversely, although coarse powders have lower surface activity, the boride phase transformation—driven by boron atom diffusion—drives the sintering densification process, and the presence of the second phase does not cause significant grain growth. This interplay between “powder surface activity,” “boride phase transformation,” and “second-phase pinning” forms a coupled relationship that is both mutually inhibitory and mutually reinforcing, collectively leading to similar sintering densification results for powders of different particle sizes.

**Figure 6:**
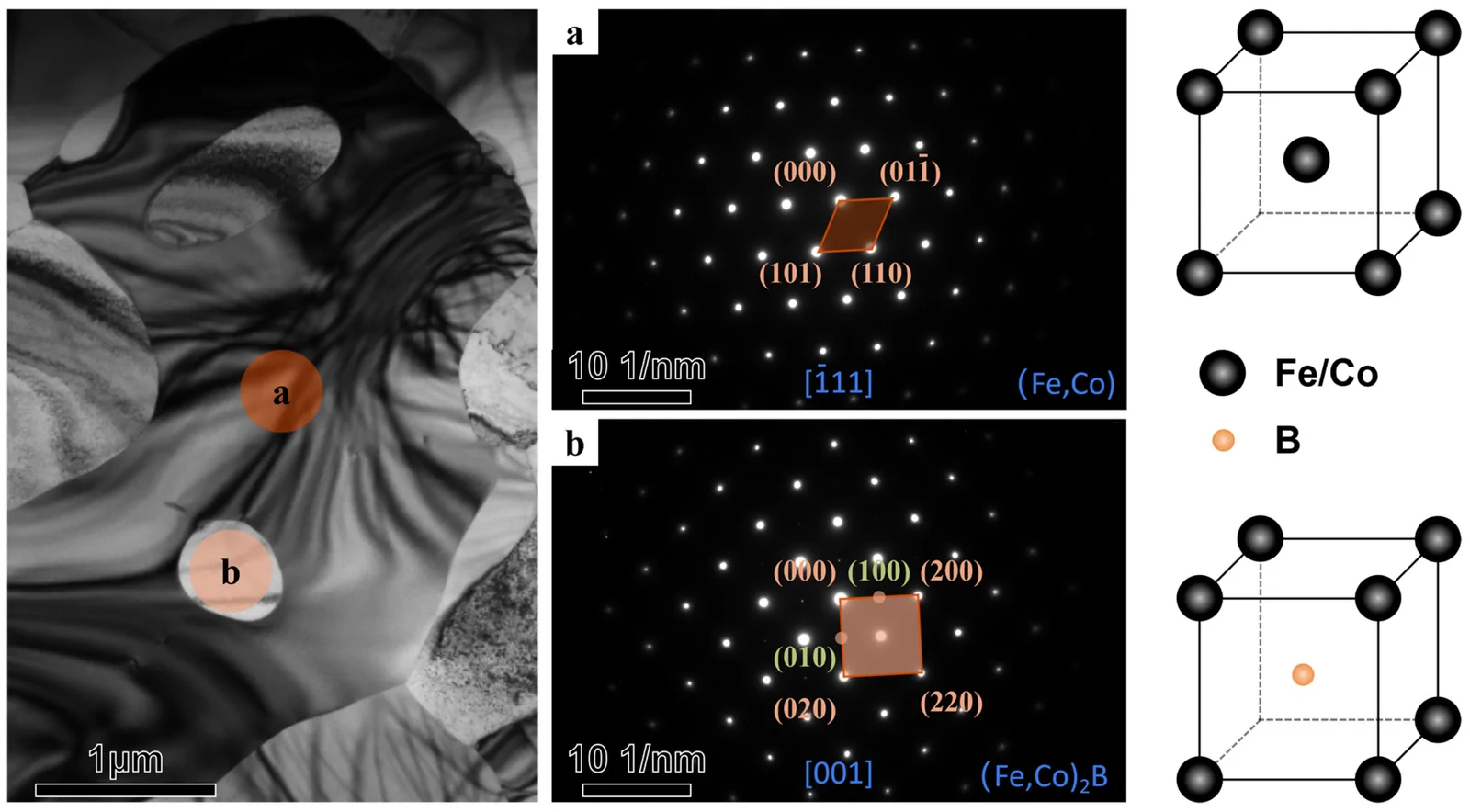
Fully transformed (**a**) (Fe,Co) phase and (**b**) (Fe,Co)_2_B phase in the sintered compact observed via transmission electron microscopy (TEM).

The TEM images and corresponding SAED patterns of the Co40Fe40B20 alloy prepared by vacuum sintering reveal the formation and growth of a new phase within an existing phase. As shown in Figure 6, the bright-field image reveals that the alloy microstructure consists of two phases: a continuously distributed dark matrix phase and a dispersedly distributed light-colored, irregularly shaped plate-like precipitate phase. The precipitate phase sizes are primarily in the range of 1–2 μm. The interface between the two phases is clear and straight, with no obvious interfacial reaction layer or compositional segregation zone.

SAED analysis was performed on the dark matrix region labeled as “a” in Figure 6, and the resulting diffraction pattern is shown in Figure 6a. The diffraction spots exhibit typical body-centered cubic (bcc) structural features [27]. As shown in Figure 6a, no system extinction spots with odd values of *h* + *k* + *l*—characteristic of the body-centered tetragonal structure (such as (100), (010)), as shown in Figure 6b. This key evidence indicates that the precipitated phase is not the thermodynamically stable I4/mcm structure Fe_2_B phase in the binary Fe-B system, but rather the (Fe,Co)_2_B phase with a simple tetragonal P4/mmm structure. A schematic diagram of its crystal structure is shown in the lower right corner of Figure 6. Fe and Co atoms occupy only lattice vertex sites, while B atoms are located at the center of the unit cell, representing a fundamental difference from the coordination environment of B atoms in the I4/mcm structure, where they occupy a trigonal prismatic interstitial site.

The lattice mismatch between the bcc-(Fe,Co) solid solution and the P4/mmm-structured (Fe,Co)_2_B phase on the (001) crystal plane is only 0.35%, enabling fully coherent growth and thereby significantly reducing the interfacial energy of the phase transformation. In contrast, the lattice mismatch between the thermodynamically stable I4/mcm-structured Fe_2_B phase and the bcc matrix is as high as 25.8%, resulting in extremely high interfacial energy and a nucleation barrier significantly higher than that of the P4/mmm structure. Therefore, during the nucleation stage, the system preferentially selects the P4/mmm structure, which has the lowest interfacial energy, for phase transformation.

The microstructure is a typical polycrystalline structure formed by sintering and recrystallization, consisting of numerous grains of varying sizes, with grain sizes ranging from approximately 2 to 8 μm, consistent with the grain size distribution observed in the sintered body via SEM and EBSD as described earlier; the grain boundaries are distinct, with no obvious subgrains or unsintered pores, further confirming that the sintered body has achieved sufficient densification and complete recrystallization. The diffraction pattern in region a is a typical single-crystal diffraction spot of a body-centered cubic (bcc) structure, directly confirming that region a consists of single-crystal grains of the bcc-(Co,Fe) matrix phase. The single-crystal particles of the second phase formed in the (Co,Fe) matrix exhibit diffraction spots characteristic of a cubic crystal system, corresponding to the (Fe,Co)_2_B boride phase. Consequently, this directly confirms that region B consists of stable (Fe,Co)_2_B boride second-phase single-crystal particles precipitated during the sintering process, further corroborating the core conclusion stated earlier: “The metastable (Fe,Co)_3_B phase in the powder is completely transformed into the (Co,Fe) phase and the stable (Fe,Co)_2_B phase upon sintering.”

Based on the gas atomization solidification theory and grain growth kinetics during sintering, this work confirms that the average matrix grain size of the sintered compact (*D*) and the median diameter of the original powder (*D*_0_) follow a power–law relationship:

(2) $$D=a\cdot D_{0}^{n}$$

where a is the pre-exponential factor, and n is the power exponent. The median particle size of the powders was measured via a laser particle size analyzer, and the average grain size of the sintered compacts was statistically calculated using the linear intercept method. After log-linear transformation of the power–law model, unary linear regression fitting was performed using the least squares method, while optimization and verification were carried out via the non-linear least squares method. The goodness of fit is:

(3) $$R^{2}=0.97$$

The final fitted equation is obtained as:

(4) $$D=0.218\cdot D_{0}^{0.563}$$

The power exponent is in excellent agreement with the theoretically derived range, and this model can accurately describe the quantitative regulation law of the original powder particle size on the grain size of the sintered compact.

## 4. Conclusions

This study systematically investigated the non-equilibrium solidification microstructural characteristics of pre-alloyed powders with different initial particle sizes (<38 μm fine powder, 38–74 μm medium powder, and 74–154 μm coarse powder) prepared by the gas atomization method, as well as the phase transformations, microstructural evolution, and macroscopic mechanical properties of the corresponding sintered bodies. Through characterization using XRD, SEM-BSE, EBSD, TEM, and performance testing, the following key conclusions were drawn:

(1) The microstructure and phase characteristics of gas-atomized powders exhibit significant particle size dependence. The cooling rate of the powder is negatively correlated with particle size. Fine powders cool rapidly and exhibit high solidification undercooling, resulting in a highly dispersed microstructure of fine dendrites and cellular crystals with low crystallinity. Element B forms a large amount of supersaturated solid solution in the (Fe,Co) matrix, and the phase composition is dominated by the metastable (Fe,Co)_3_B boride phase. As the powder particle size increases, the cooling rate decreases, enabling the dendrites to fully grow into radially arranged columnar dendrites. This results in increased crystallinity and reduced non-equilibrium, with a slight increase in the proportion of the supersaturated (Fe,Co) solid solution phase.

(2) During the sintering process, the alloy undergoes complete phase transformation and microstructural homogenization. After sintering, the metastable (Fe,Co)_3_B phase formed by non-equilibrium solidification completely decomposes in all powder sizes, undergoing the phase transformation reaction (Fe,Co)_3_B → (Fe,Co)_2_B + (Fe,Co), ultimately forming an equilibrium two-phase microstructure with (Co,Fe) as the matrix and (CoFe)_2_B as the second phase. This result was directly verified at the crystallographic level through XRD, EBSD, and TEM analysis; simultaneously, the sintering and recrystallization process eliminated some of the dendritic solidification texture present in the powder state, forming an equiaxed grain structure, although coarse powders retain some dendritic characteristics.

(3) The particle size of the original powder significantly influences the microstructural uniformity of the sintered body. Fine powder corresponds to a sintered body with a fine and uniform (Fe,Co) matrix, with an average grain size of approximately 1.2–1.8 μm. The (Fe,Co)_2_B second phase is dispersed along the matrix grain boundaries and within the grains, exerting a strong Zener pinning effect on the grain boundaries, which effectively suppresses grain growth. As the initial powder particle size increases, the matrix grains and the second phase in the sintered body gradually coarsen. Coarse powder corresponds to a sintered body with an average matrix grain size of 3–5 μm, where the second phase forms a semi-continuous network structure along the grain boundaries, resulting in a significant decrease in microstructural uniformity. Fitting of experimental data revealed a power–law relationship between the matrix grain size of the sintered body and the initial powder particle size, expressed as $D=a\times D_{0}^{0.563}$, which accurately describes the regulation mechanism of powder particle size on the growth of sintered grains.

The sintered bodies prepared from the three sets of powders with different particle sizes all achieved high densification, with no significant differences in macroscopic properties. The densities of the sintered bodies corresponding to the fine, medium, and coarse powders were 7.85 g/cm^3^, 7.86 g/cm^3^, and 7.88 g/cm^3^, respectively, with a maximum relative deviation of less than 0.4%; the Vickers hardness values were 682 HV, 689 HV, and 683 HV, respectively, with a maximum relative deviation of approximately 1.0%. These performance variations fall within the standard margin of error for material testing. This phenomenon stems from the Hall–Petch fine-grain strengthening effect resulting from grain refinement, which couples and offsets the weakening effect caused by second-phase coarsening, ultimately resulting in sintered bodies prepared from powders of different particle sizes achieving the same degree of densification and hardness.

## Data Availability

The original contributions presented in this study are included in the article. Further inquiries can be directed to the corresponding authors.

## References

[1] Cestarollo L., Srinivasan K., El-Ghazaly A. (2022). Investigation of perpendicular magnetic anisotropy in Pt/Co_20_Fe_60_B_20_/Pt multi-layer structures. J. Magn. Magn. Mater..

[2] Chen Y.-T., Hsieh W.H. (2013). Thermal, magnetic, electric, and adhesive properties of amorphous Co_60_Fe_20_B_20_ thin films. J. Alloys Compd..

[3] Choi G.-M. (2020). Exchange stiffness and damping constants of spin waves in CoFeB films. J. Magn. Magn. Mater..

[4] Dwivedi J., Gupta M., Reddy V.R., Mishra A., Srihari V., Pandey K.K., Gupta A. (2018). Effect of heavy metal interface on the magnetic behaviour and thermal stability of CoFeB film. J. Magn. Magn. Mater..

[5] Arif M., Zhang X., Amir M., Liu E., Xu F. (2023). Tailoring the magnetism and spin dynamics in CoFeB thin films by post annealing for spintronics applications. J. Mater. Sci. Mater. Electron..

[6] Seeger R.L., Millo F., Mouhoub A., de Loubens G., Solignac A., Devolder T. (2023). Inducing or suppressing the anisotropy in multilayers based on CoFeB. Phys. Rev. Mater..

[7] Zhong Z., Ye J., Wang Y., Ren Y., Zhang J., Zhang B., Ye F. (2025). Densification kinetics, microstructural evolution, and mechanical properties of (TiB+ TiC+ Ti_3_Si)/TC4 composites under pressureless sintering. J. Alloys Compd..

[8] Liu Y.Q., Zhao X.S., Yang J., Shen J.Y. (2011). Thermodynamic optimization of the boron–cobalt–iron system. J. Alloys Compd..

[9] Lu L.L., Lin Y., Dai J., Yan Q., Xu Q.H., Chen F.G., Jain A., Wang Y.G. (2023). Co-evolution of the atomic/nano scale structural heterogeneities and magnetic properties of Fe-Si-B-Cu metallic glass during relaxation with composition fluctuations. J. Non-Cryst. Solids.

[10] Yue X., Wang W., Ru H., Zhang C., Zhao Y., Sun S., Xia Q. (2022). Effect of boron carbide particle size distribution on the microstructure and properties of reaction bonded boron carbide ceramic composites by silicon infiltration. J. Inorg. Mater..

[11] Wu Y., Skokov K.P., Schäfer L., Maccari F., Xu H., Wang X., Jiang C., Gutfleisch O. (2024). A systematic investigation of Pr-rich Pr-(Fe, Co)-B material system: Phase formation, microstructure and magnetic property. Acta Mater..

[12] Wang L., Zheng Z., Chen Y., Chen X., Qiu Z., Zeng D., Yuan S. (2023). The influence of Co on the magnetic properties of Fe–Si–B–Nb–Cu system. Phys. B Condens. Matter.

[13] Pan Y., Huang L., Zhang J., Du Y., Luo F., Zhang Y. (2024). Study on microstructures, properties and mechanisms of sinter-joining refractory metal W30Si powders and sputtering targets by spark plasma sintering technique. Int. J. Refract. Met. Hard Mater..

[14] Duan X., Ju S., Li Y., Zhu Z., Zhang H., Zhang W. (2023). Effects of B/P and Co/Fe substitutions on glass-forming ability and soft magnetic properties of a Fe_80_P_13_C_7_ metallic glass. J. Non-Cryst. Solids.

[15] Huang Y., Li H., An X., Quan W., Ji C., Zheng Q., Du J. (2025). Optimized microstructure and improved magnetic properties of sintered Nd-Fe-B magnets induced by co-doping high and low melting point elements. J. Alloys Compd..

[16] Li M., Wang S., Zhang S., Fang S., Yu G. (2019). The perpendicular magnetic anisotropies of CoFeB/MgO films with Nb buffer layers. J. Magn. Magn. Mater..

[17] Morgunov R.B., Koplak O.V., Allayarov R.S., Kunitsyna E.I., Mangin S. (2020). Effect of the stray field of Fe/Fe_3_O_4_ nanoparticles on the surface of the CoFeB thin films. Appl. Surf. Sci..

[18] Rana B., Miura K., Takahashi H., Otani Y. (2020). Underlayer material dependent symmetric and asymmetric behavior of voltage-controlled magnetic anisotropy in CoFeB films. J. Phys. Condens. Matter.

[19] Chen Z., Zhang K., Zhu Q., Guo Q., Jiang Y. (2021). Microstructure and phase dependence of magnetic properties of CoFeB nanowires. Vacuum.

[20] Silva A.S., Sá S.P., Bunyaev S.A., Garcia C., Sola I.J., Kakazei G.N., Crespo H., Navas D. (2021). Dynamical behaviour of ultrathin [CoFeB (t CoFeB)/Pd] films with perpendicular magnetic anisotropy. Sci. Rep..

[21] Lin N., Lin Y., Wu G., Chen X., Zhang Z., Hu X., Zhuang N. (2021). Growth of high quality Si-based (Gd_2_Ce)(Fe_4_Ga) O_12_ thin film and prospects as a magnetic recording media. J. Alloys Compd..

[22] Bottcher T., Lee K., Heussner F., Jaiswal S., Jakob G., Klaui M., Hillebrands B., Bracher T., Pirro P. (2021). Heisenberg exchange and Dzyaloshinskii–Moriya interaction in ultrathin Pt (W)/CoFeB single and multilayers. IEEE Trans. Magn..

[23] Xu Z., Qin L. (2021). Effects of sputtering parameters and annealing temperatures on magnetic properties of CoFeB films. J. Magn. Magn. Mater..

[24] Florez S.H., Papusoi C., Mani P., Desai M. (2021). Microstructural segregation in high anisotropy [Co/Pt] n superlattice films. J. Magn. Magn. Mater..

[25] Liu X.B., Nlebedim I.C. (2024). Robustness of magnetocrystalline anisotropy and coercivity in Fe–Co–B. Phys. B Condens. Matter.

[26] Raghavan V. (2012). B-Co-Fe (boron-cobalt-iron). J. Phase Equilibria Diffus..

[27] Sunbul S.E., Akyol S., Onal S., Ozturk S., Sozeri H., Icin K. (2023). Effect of Co, Cu, and Mo alloying metals on electrochemical and magnetic properties of Fe-B alloy. J. Alloys Compd..

[28] Du W., Liu M., Wang G., Su H., Liu B., Meng H., Tang X. (2022). Stack structure and CoFeB composition dependence of perpendicular magnetic anisotropy employing Pt heavy metal layer. J. Alloys Compd..

[29] Das C., Alagarsamy P. (2018). Tuning the magnetic properties of stripe domain structured CoFeB films using stack structure with spacer layer thickness dependent interlayer coupling. J. Magn. Magn. Mater..

[30] Gupta N., Gupta M., Kumar D., Rai S., Gupta P. (2024). Tailoring thermal stability and in-plane magnetic anisotropy of CoFeB alloy thin films via growth conditions engineering. Intermetallics.

[31] Liang Y., Yang M., Kong D., Huang T., Wang J., Yang Z., Zheng B., Song P. (2024). Effect of annealing on corrosion resistance and mechanical properties of Cr–Fe–Ni–Co–Mo–Si–B amorphous coating. J. Mater. Res. Technol..

[32] Cheng T., Yang Z., Zhang X., Yu G. (2025). The physical properties of iron-rich Fe-Co alloys: Analysis from the perspective of Co concentration. Phys. Lett. A.

[33] Bazlov A.I., Milkova D.A., Zanaeva E.N., Strochko I.V., Tabachkova N.Y., Inoue A. (2024). Formation, thermal stability and soft magnetic properties of Fe-Co-B-Si amorphous alloys with ultrahigh saturation magnetic induction of 2.0 T. J. Alloys Compd..

[34] Lin B., Han Y., Zuo D., Wang N., Zhang Y., Fu H., Gao C. (2026). An RSM-Based Investigation on the Process–Performance Correlation and Microstructural Evolution of Friction Stir Welded 7055 Al/2195 Al-Li Dissimilar T-Joints. Materials.

